# Dermatology mycology diagnostics in Ireland: National deficits identified in 2022 that are relevant internationally

**DOI:** 10.1111/myc.13549

**Published:** 2022-12-09

**Authors:** James Powell, Emma Porter, Siobhan Rafferty, Sinead Field, Nuala H. O'Connell, Colum P. Dunne

**Affiliations:** ^1^ Department of Microbiology University Hospital Limerick Limerick Ireland; ^2^ School of Medicine and Centre for Interventions in Infection, Inflammation, and Immunity (4i) University of Limerick Limerick Ireland; ^3^ Department of Dermatology University Hospital Limerick Limerick Ireland

**Keywords:** Dermatomycoses, dermatomycosis, diagnostics, mycology, PCR, tinea

## Abstract

**Background:**

Conventional testing methods for dermatophytes are time‐consuming, and resource limitations in our institution have prompted curtailed access to these diagnostics.

**Objectives:**

Evaluation of our hospital's dermatological mycology diagnostic services and similar services nationally.

**Methods:**

This was a retrospective observational study on skin, hair and nail mycology samples in our institution comparing twenty five‐year periods (2011–2015 and 2016–2021), including analysis of dermatology clinic data and correspondence related to fungal infection. A survey of national public hospitals' laboratories was conducted to evaluate their mycology testing capabilities.

**Results:**

The total 5 year test count prior to curtailment was 4851 specimens comprising 90% (*n* = 4344) from general practice and 6% (*n* = 290) from dermatology clinics. For the 5 years post curtailment, 64.5% (582/903) of specimens were from dermatology clinics. Dermatology clinic data demonstrated doubling of attendances (for all conditions) and of correspondence related to fungal infection. During this time also, national dermatological antifungal purchasing increased 11%. Ten of 28 Irish public hospital laboratories reported the provision of in‐house dermatological mycology testing, and none had routine availability of susceptibility or molecular testing of dermatophytes.

**Conclusion:**

This study is the first to report an appraisal of dermatological fungal diagnostic services in Ireland. Insufficient testing capacity implies that patients are either being treated for fungal infection without appropriate diagnostic confirmation, or being left untreated because of the lack of access to diagnostics. The introduction of molecular detection methods and susceptibility systems would enhance testing capabilities and reduce the requirement for the external referral.

## 1 INTRODUCTION

Dermatophyte infections are among the most common global diseases, affecting 25% of the world's population, with asymptomatic carriage in 30%–70% of adults.^1^ Moreover, in the last two decades, there has been a dramatic increase in their incidence, due to a range of factors including socioeconomic problems, international travel, immigration from tropical countries and contact with animals, particularly pets.^2^ The clinical features of dermatophytosis may be mistaken for a wide range of other dermatological diseases including bacterial folliculitis, psoriasis and eczema.^3^ Many localised uncomplicated fungal skin infections in healthy individuals can be treated effectively by community pharmacists and general practitioners, however, access to accurate pathogen identification is important in moderate to severe disease, complicated or recalcitrant disease; in order to direct treatment appropriately. For example, in tinea capitis, treatment is often commenced based on clinical diagnosis; however, the choice of oral antifungal agent is dependent on the suspected species and subsequent pathogen identification; also, guidelines recommend that the definitive end point for adequate treatment must be the mycological cure, rather than clinical response.^4^ Infections with anthropophilic species such as *Trichophyton violaceum* or *Trichophyton soudanense* have shown a good response to terbinafine, yet zoophilic pathogens such as *Microsporum canis* have better cure rates with the use of griseofulvin or itraconazole.^5^, ^6^

Historically there has been a preponderance of zoophilic dermatophytes in our region,^7^ but a recent epidemiological study demonstrated a shift in prevalence to predominantly anthropophilic species over a 20‐year period.^8^ In the latter period of this study, mycology testing of skin, hair and nail samples was outsourced, and access was curtailed for patients in primary care settings. Conventional mycological diagnostic methods are time‐consuming,^3^ and when faced with staff shortages in 2016, mycology testing was outsourced to a referral laboratory. Thereafter, requests for fungal testing of skin, hair and nail samples were restricted to consultant dermatologists and other practitioners with specialist training, reducing the number of tests performed.

The shortage of medical laboratory scientists is neither a recent phenomenon nor is it simply a local problem for our laboratory; calls to action to address the shortage began in the 1980s,^9^, ^10^ and even prior to the COVID‐19 pandemic it was recognised that the number of new medical laboratorians entering the workforce was not keeping up with future demand.^11^ In a National survey of the United States of America in 2018, vacancy rates in laboratories were ‘considerably higher’ than a similar survey in 2016, and Microbiology Departments were amongst the worst affected with vacancy rates over 10%.^12^ At the time of writing, in our Microbiology Department we have a vacancy rate of 21%, and this has been an on‐going issue for many years.

In the scientific literature, there is an abundance of guidance^4^, ^13^, ^14^, ^15^ and recommendations^16^, ^17^, ^18^, ^19^, ^20^, ^21^, ^22^, ^23^, ^24^, ^25^, ^26^, ^27^ for the diagnosis of dermatomycoses and onychomycoses. However, little is known of the degree to which laboratories have adopted new technologies such as molecular identification tests and antifungal susceptibility testing of dermatophytes. Ireland has no national mycology reference laboratory and fungal skin, hair or nail infections are not notifiable diseases, so there is no oversight or co‐ordinated approaches to diagnosis and surveillance of these pathogens or their susceptibility to anti‐fungal agents. The aim of this study is to perform an evaluation of the dermatological mycology diagnostic service of our hospital and the other hospitals of Ireland, in comparison to similar services internationally, and recognised best practice.

## 2 METHODS

### 2.1 Ethics statement

This study was approved by the Research Ethics Committee of University Limerick Hospital Group, Limerick, Ireland.

### 2.2 Setting

The Department of Clinical Microbiology at University Hospital Limerick (UHL) provides a centralised microbiology service for six acute hospital sites of the region's hospital group, University of Limerick Hospitals' Group (ULHG). This service is provided to public and private healthcare facilities in the region including general practice, for a population of circa 400,000 people. Of note, there are no electronic patient records in this group of hospitals. Previous related research from our institution includes fungal bloodstream infections in our ICU patients,^28^ an epidemiological analysis of dermatomycoses and onychomycoses in our region over a period spanning 20 years,^8^ and several reports of multi‐resistant organisms detected in our hospitals, many of which resulted in outbreaks.^29^, ^30^

### 2.3 Data and Analysis

All mycology laboratory test counts from January 2001 to December 2021 were extracted from the Laboratory Information Management System (LIMS, iLab, Dedalus Healthcare, Italy), to provide an historical context to recent trends in the numbers of tests performed. For the period 2011–2021, a data extract of dermatology clinic attendance figures for the hospital was performed from the patient management system (iPMS, Dedalus Healthcare, Italy). Figures for the five‐year periods prior to and following July 2016 (when the change to testing methodology was implemented) were recorded. Similarly, a keyword search term count was performed of the patient clinical letters database (Filemaker Pro, Claris International) held at the dermatology clinic. The keywords ‘fungal’, ‘tinea’ and ‘onychomycosis’ were searched for in the letters of correspondence sent to general practitioners. The count of letters containing these keywords allowed a crude comparison to be made of the number of patients with these conditions seen in the periods before and after access to diagnostics was restricted.

A survey was performed in all of the 28 public hospital Microbiology laboratories of Ireland to determine how many of those laboratories performed in‐house mycology testing of skin, nail and hair samples, and which of them routinely performed polymerase chain reaction (PCR) and/or susceptibility testing of dermatophytes and non‐dermatophyte moulds. The respondents were invited to supply test count data if they wished. This survey took the form of an e‐mail request in January 2022 and subsequent follow‐up of non‐responders.

The pharmaceutical suppliers of the main dermatological anti‐fungal agents were contacted by e‐mail in January 2022, with follow‐up e‐mails for non‐responders. The companies were asked for details of the number of their unit sales per product for the Irish state and/or for the Mid‐West region of Ireland, especially data from 2011 to 2021 where possible. The companies were Brown and Burk IR Limited, GlaxoSmithKline Consumer Healthcare (Ireland) Limited, Novartis Ireland Limited, Viatris Global Healthcare T/A Mylan Limited, Johnson & Johnson Limited and Janssen Sciences Ireland.

Data were analysed using Microsoft Excel.

## 3 RESULTS

**FIGURE 1:**
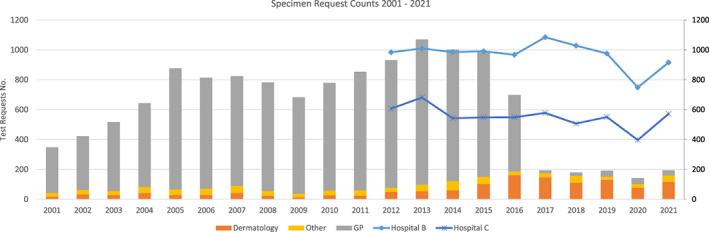
Number of specimen requests per requesting location 2001–2021. Data are also shown for two other hospitals in Ireland, ‘Hospital B’ which is located in the south of the country, and ‘Hospital C’ which is in the East.

For the five‐year period 2011–2015, the median number of skin, hair and nail specimens for mycology analysis received in our laboratory from general practitioners (GPs) was 855 specimens per annum. For the corresponding period following the restriction of access to this service (2017–2021), the median test count was 35 specimens per annum (i.e., a 96% reduction). The positivity rate (microscopy and/or culture) of these samples increased from 36.5% to 40% across these two periods. The dermatology clinic of our hospital showed an increase from 54 specimens per annum to 117 specimens per annum (117% increase) for the same two time periods and a reduction in the positivity rate from 30% to 27%. See Figure 1 for a chart of specimen requests per requesting location.

**FIGURE 2:**
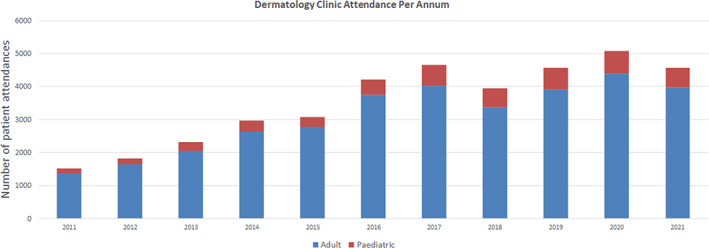
Annual attendance figures at the Dermatology Clinic, University Hospital Limerick.

Total dermatology clinic attendance figures showed a similar increase over the two time periods. The median annual attendance for the clinic in the pre‐curtailment period was 2320 and the corresponding figure post‐curtailment was 4570 attendances (97% increase). This increase was weighted more heavily in favour of paediatric patients (140% increase) rather than adult patients (94% increase). See Figure 2 for a chart of annual attendance figures at the clinic.

**FIGURE 3:**
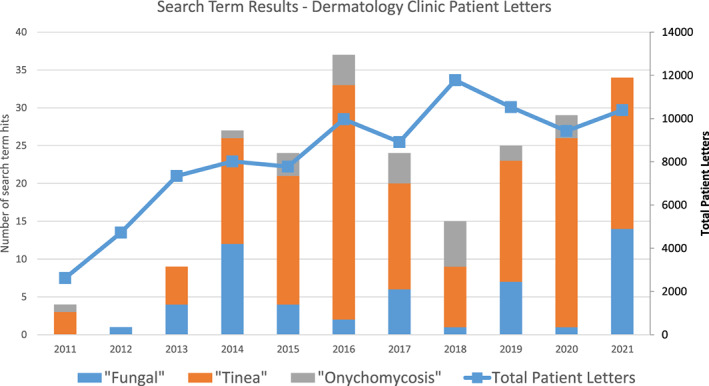
Count of search term matches in the Filemaker Pro patient letters database of the Dermatology Clinic at University Hospital Limerick. Counts of search term matches are displayed in bars corresponding with the primary vertical axis, total letter counts correspond with the figures on the secondary vertical axis (on the right).

The results for the count of letters from the patient letters database of the dermatology clinic with matches for the specific search terms ‘fungal’, ‘tinea’ and ‘onychomycosis’ also showed an increase. The total number of letters per annum in the pre‐curtailment period was 65 letters (21 ‘fungal’, 39 ‘tinea’ and 5 ‘onychomycosis’), and there were 127 letters per annum (29, 83 and 15, respectively) in the post‐curtailment period – a 95% increase. See Figure 3 for the number of matches for patient letters containing the search terms ‘fungal’, ‘tinea’ and ‘onychomycosis’, as well as the total number of patient letters recorded per annum in the clinic.

**FIGURE 4:**
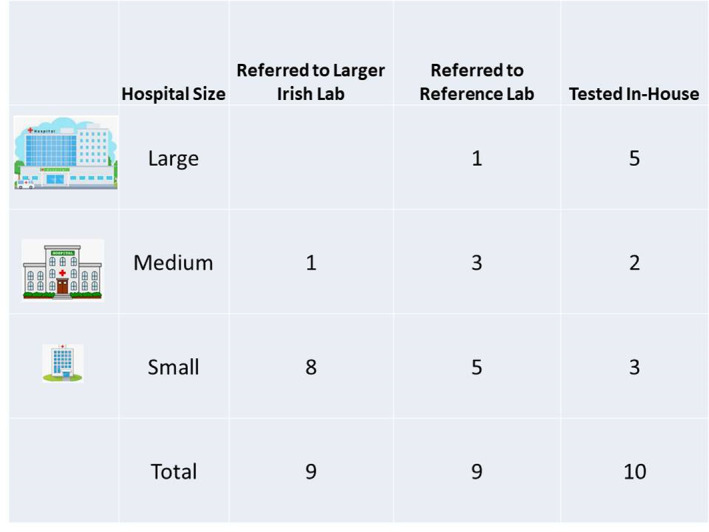
Survey results of Irish Public Hospital Laboratories provision of fungal testing of skin, hair and nail samples.

In January to March 2022, a survey of the Microbiology laboratories of the public health service system (Health Services Executive) hospitals in the Republic of Ireland revealed that 10 of the twenty‐eight laboratories continue to perform in‐house fungal testing of skin, hair and nail samples. See Figure 4 for a chart of the results of this survey. Nine laboratories refer their specimens to laboratories in larger hospitals in their region, often as part of a hub‐and‐spoke service that applies to many of the more specialised microbiology tests. Nine other laboratories refer their samples to a private reference facility for testing. Our laboratory was the only one of the six large (>600 beds) hospitals which did not provide in‐house testing of these samples. Medium‐sized hospitals were defined for this study as those accommodating 300–600 beds, and small hospitals were those with <300 beds. The bed capacity provides only a very rough estimate of the testing throughput of the laboratories; much of the testing workload comes from community healthcare facilities and general practice, which can vary widely for each hospital depending on their location.

The laboratories were also asked whether they had curtailed access to their fungal testing of skin, hair and nails, and were invited to supply test count data. Two hospitals supplied 10 years of test count figures, one from the south of the country (‘Hospital B’) and one from the east of the country (‘Hospital C’), neither of which has had to restrict access to mycology diagnostic services, see Figure 1 for details. Some laboratories reported that they did not provide microscopy results for some of their users (usually general practitioners), but access to fungal culture testing was only restricted by two laboratories (including ours). The second laboratory introduced this measure as a result of the surge in workload due to the Covid‐19 testing. In a follow‐up question to the above survey, the respondents were asked whether they had in‐house capability for either susceptibility testing or PCR testing of dermatophytes. One of the respondents had validated a PCR system but had not yet brought it into routine use, and two other hospitals had trials of systems in progress. As such, at the time of the survey there were no hospitals in Ireland with a dermatophyte PCR system available for routine use. None of the respondents had a susceptibility testing system in use, and since there is no national reference lab facility in Ireland, isolates would need to be sent to the United Kingdom for susceptibility testing if required.

In February 2022, the following pharmaceutical companies were contacted for sales data (11 years of data if possible) on their dermatological anti‐fungal products, and their responses are included below:

1. Brown and Burk IR Limited (oral griseofulvin): No response.
2. GlaxoSmithKline Consumer Healthcare (Ireland) Limited (topical terbinafine): No data available.
3. Novartis Ireland Limited (oral terbinafine): Data for 2017–2021 supplied.
4. Viatris Global Healthcare T/A Mylan Limited (oral terbinafine): Data for 2018–2021 supplied.
5. Johnson & Johnson (Ireland) Limited (topical miconazole, topical clotrimazole, topical ketoconazole, topical terbinafine): Data for 2017–2021 supplied.
6. Janssen Sciences Ireland (topical miconazole and hydrocortisone): Data for 2017–2021 supplied.

**FIGURE 5:**
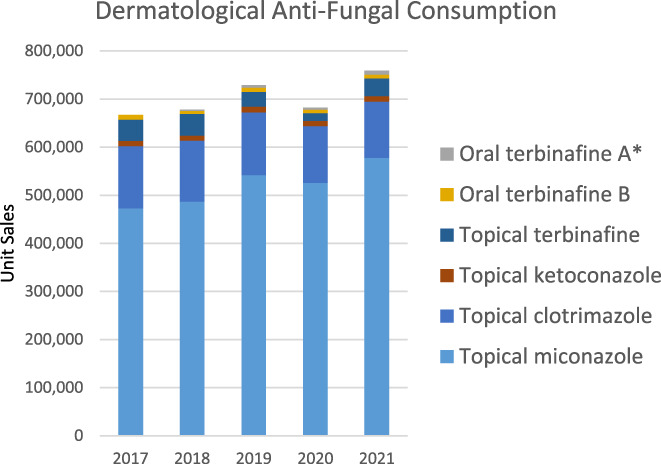
Unit sales volumes of common anti‐fungal skin preparations and oral agents. *Data for 2018–2021 only.

No data prior to 2017 were available, but the data provided for the period 2017–2021 (excluding Nailderm tablets) showed a 12.5% increase in product sales. The data for 2018–2021 (including Nailderm tablets) showed an 11% increase in product sales. Figure 5 provides a chart of the volume of sales for each of the above products.

## 4 LIMITATIONS

Local pharmacy sales data were not available for the study. The Primary Care Reimbursement Service (PCRS) was contacted for antifungal reimbursement claims data. No data were available by the time the study concluded.

## 5 DISCUSSION

The incidence of fungal skin infections is increasing at an alarming rate worldwide.^31^ Increased incidence was demonstrated in our region by surrogate measures that were examined in this study: Anti‐fungal sales data and dermatology clinic records of confirmed or suspected infections both show double‐digit increases in the last 5 years. Furthermore, the twenty‐year records of test requests of skin, hair and nail samples show year‐on‐year increases right up to the point when access to testing was curtailed. It is evident from our patient letter counts (see Figure 3) that patients with fungal‐related disorders represent a very small proportion of cases seen at our dermatology clinics, suggesting the main burden of disease and treatment management is in the community setting by general practitioners and pharmacists with curtailed access to appropriate mycological investigations. Reports of outbreaks involving dermatophytes are commonplace in the scientific literature; a PubMed search for ‘tinea’ and ‘outbreak’ for 2012–2021 provides 767 results. Tinea unguium or onychomycosis was the most common body site mentioned in the study title (50.3% of those with a site stated in the title, 172/342), followed by tinea capitis (27.8%, *n* = 95), tinea versicolor/corporis (7.9%, *n* = 27) tinea pedis (7.3%, *n* = 25) and tinea faciei (2.3%, *n* = 8). Where a geographical region is mentioned in the title (*n* = 423), regions in Asia were the most common (24.8%, *n* = 105), followed by Africa (24.3%, *n* = 103), Europe (20.8%, *n* = 88), the Middle East (13/7%, *n* = 58), North and South America (4.7% and 10.2%, respectively) and Oceania (1.4%, *n* = 6). No recent reports of outbreaks are available from Ireland, although a study from Dublin in 2006 described a disproportionate (85.5%) number of patients of African extraction among their paediatric tinea capitis patients.^32^ Fungal outbreaks are not unknown on this island however; in 1948, a cluster of 368 tinea capitis cases were detected.^33^ Despite this, dermatophyte infections are not listed as a notifiable disease in this country, so there is no obligation to report them. A considerable shift in the epidemiology of dermatophytes has been demonstrated in our region in the last 20 years, with an increasing proportion of anthropophilic species detected from both skin or hair samples and from nail samples,^8^ and this has been mirrored in many other countries.^34^, ^35^, ^36^, ^37^ The migration of people, children in particular, during wartime has been linked with an increase in dermatomycoses. This has been reported in the former Yugoslavia during the war that took place there in the 1990s, and was previously reported after the second world war, when dermatomycoses spread epidemically.^38^ At the time of writing, more than 14 million people have fled Ukraine due to the war taking place there,^39^ many of them women and children. It is important now more than ever that dermatomycoses are monitored and identified to prevent large outbreaks from occurring.

The results of this study demonstrated a dramatic reduction in the processing of specimens for fungal analysis from GPs after curtailment of mycology diagnostic services. The corresponding increase in dermatology clinic samples did not fill the gap left by this drop in community specimens, which could be explained in part by patients being treated for fungal infection without appropriate diagnostic confirmation, or being left untreated because of the lack of access to diagnostics. Clinical papers^24^, ^25^, ^26^ and dermatology guidelines^13^ unanimously call for laboratory confirmation of fungal infection before oral treatment of onychomycosis is started. Clinical findings, nail disease pattern and mycological investigations are important in the treatment of onychomycosis (fungal nail disease); in particular topical treatments are most effective in superficial onychomycosis but often ineffective in subungual or dystrophic onychomycosis with prolonged courses of systemic antifungals required to eradicate infection.^40^ Additionally, diagnosis of onychomycosis can be challenging, with similar clinical features seen in non‐dermatophyte nail infections, and non‐infectious conditions such as psoriasis, chronic trauma, lichen planus and nail bed malignancies.^41^ Antifungal medications are known to have multiple potential side effects and drug interactions, so prolonged courses in the absence of dermatophyte confirmation is not advised. Mycological identification not only supports diagnosis, it influences antifungal therapy choice and in select cases provides susceptibility information for recalcitrant infections.^13^, ^42^ Despite the recommendations for microbiological confirmation, investment in fungal diagnostics in this country has been poor. Just 10 of the twenty‐eight laboratories surveyed had in‐house mycology testing capabilities for skin, hair and nail samples, and none reported access to in‐house PCR or susceptibility testing of dermatophytes.

Antifungal resistance has been called a ‘global public health threat’.^43^, ^44^ This is exemplified in India where terbinafine resistant *T*. *indotineae* are highly prevalent,^45^ and terbinafine resistance in dermatophytes has also been reported in Iran, Japan, Denmark, Belgium, Finland, Switzerland, Germany, the United States, Canada, Bahrain and Brazil.^44^ Terbinafine resistance is especially concerning because alternative therapeutic options to treat dermatophytoses are limited. Antifungal resistance is also probably underestimated, since many countries,^44^ including our own, have not been performing susceptibility testing. Susceptibility testing of dermatophytes isolated from recalcitrant infections is imperative,^19^, ^22^, ^46^, ^47^ so access to this capability should be a priority for our diagnostic services. This would be most readily achieved by the creation of a mycology reference lab for the country, a resource that no national health service should be without. For such testing, current practice in our institution is the transfer of samples overseas to The Mycology Reference Laboratory in Bristol, United Kingdom. This further compounds costs to the health service and delays timely diagnosis.

MALDI‐TOF MS (matrix‐assisted laser desorption/ionisation time‐of‐flight mass spectrometry) instrumentation has been reported to be capable of identifying dermatophytes,^23^, ^25^ although not yet to the same level of accuracy achieved by conventional methods. The availability of these instruments in most modern microbiology laboratories may mean that in the future the identification of fungal pathogens may not need to be a laborious and subjective methodology, and should make it easier for smaller laboratories to implement mycology testing without the specialised knowledge and experience required to identify fungi visually (macroscopically and microscopically).

Nucleic acid amplification tests have replaced many of the conventional diagnostic techniques of the microbiology laboratory, and mycology testing is no exception. The poor sensitivity of microscopy and culture, particularly after the onset of empirical treatment^21^ and the long turnaround time for culture results give PCR testing a distinct advantage over traditional methods. There is an abundance of published research available evaluating dermatophyte PCR systems, but consensus has yet to be achieved on their applicability. The earliest publications^48^, ^49^ described PCR as a supplement to culture for assisting organism identification; later publications^5^ give a more prominent role to PCR but still suggest that classical methods ‘are still warranted for training purposes and when encountering specific diagnostic problems’. More recently however we see a publication^50^ suggesting that PCR can ‘replace microscopy and culture for routine dermatophyte diagnosis’, but another author^51^ regarding the same PCR platform says that direct microscopy ‘remains relevant’ for these specimens. The Netherlands National Healthcare Institute report a higher predictive value for the PCR test over direct microscopy and culture, and they recommend that it should therefore replace traditional diagnostics in routine care.^20^ Many in‐house PCR systems have been developed, some even achieving ISO 15189 accreditation,^52^ but the most straightforward process for introducing a PCR system is via a ‘CE‐IVD’ marked commercial kit; some commercial kits are available that have not been fully evaluated (‘research use only’), these should not be used for routine diagnosis. There are four ‘CE‐IVD’ marked dermatomycosis PCR platforms from three manufacturers available for use in Ireland currently: ‘Dermagenius® 2.0’ and ‘Dermagenius® 3.0’ (Pathonostics®), ‘EUROArray Dermatomycosis’ (EUROImmun) and ‘Dermatophytes and Other Fungi 12‐Well’ (AusDiagnostics). All four platforms have been described previously.^21^, ^44^, ^50^, ^51^, ^53^, ^54^, ^55^, ^56^, ^57^ See Table 1 for a summary of the dermatological fungal isolates captured by each of these kits, and a full list of targets is available in the Appendix S1. Details are also available in the Appendix S1 for another kit which is currently marked ‘Research Use Only’: ‘Novaplex^tm^ Dermatophyte Assay’ (Seegene).

**TABLE 1:** ‘CE‐IVD’ marked commercial dermatomycosis PCR kits available in Ireland.

		Dermagenius® 2.0	Dermagenius® 3.0	EUROArray dermatomycosis	Dermatophytes and other fungi
		CE–IVD	CE–IVD	CE–IVD	CEIVD
	ULHG 02–21	Pathonostics®	Pathonostics®	EUROImmun	Ausdiagnostics
		Netherlands	Netherlands	Germany	Australia
Dermatophytes	2246	99.7%	100.0%	100.0%	99.9%
Non‐derm mould	350	0.0%	34.9%	35.1%	9.7%
Candida	455	28.4%	58.9%	76.9%	77.8%
Total	3051	77.6%	86.4%	89.1%	86.2%

*Note*: ‘ULHG’02–21 figures are the numbers of detections in total in UL Hospitals Group from 2002 to 2021. The percentages given are the proportion of isolates within each category that were matched with targets declared by the kit manufacturers.

Other studies have shown that the widespread use of over‐the‐counter antifungals may be promoting resistance, most notably to the azole drugs, which can mean that oropharyngeal, vaginal or even systemic yeast infections may need to be treated with less desirable alternatives such as amphotericin B with possible complications and renal toxicity.^58^ The increased use of immunosuppressive therapy means that invasive fungal infections are an emerging problem worldwide, and the incidence of azole resistance is increasing.^59^, ^60^ Our data show significant use of topical azole creams and powders in this country; over 700,000units purchased by a population of 5 million people in 2021.

Dermatological mycology testing has not been prioritised in many laboratories around the world, including our own, yet there is growing international evidence of increased incidence of infections and resistance to anti‐fungal agents. In Ireland, we have a growing population and increasing immigration, yet the testing capacity of our laboratories are being curtailed, susceptibility testing of dermatophytes is not being performed and new technologies have not been adopted. Furthermore, there is suboptimal epidemiological tracking of organisms and their antifungal susceptibilities, and there is no national oversight. Dermatological fungal infections are commonly misconceived as a cosmetic problem, but left untreated they can cause pain, physical impairment, increased risk of infections such as cellulitis and osteomyelitis in immunocompromised or diabetic patients, and a significant negative impact on quality of life.^18^ Recently, the WHO published a list of fungal priority pathogens causing systemic invasive infections, in this report, they suggest that future reports will include those causing dermatomycoses, highlighting the economic and health impact of the same. This study serves to highlight the need for improvement of current national practices in dermatological mycology testing, and proposes practical steps toward improving them.

## AUTHOR CONTRIBUTIONS

JP: Conceptualisation (equal); Writing–original draft (lead); Data curation (lead); Methodology (lead); Writing–review and editing (equal). EP: Conceptualisation (equal); Writing–original draft (supporting); Writing–review and editing (equal); SR: Writing–original draft (supporting); Writing–review and editing (equal). SF: Conceptualisation (equal); Writing–original draft (supporting); Writing–review and editing (equal). NOC: Conceptualisation (equal); Writing–original draft (supporting); Writing–review and editing (equal). CPD: Conceptualisation (equal); Writing–original draft (supporting); Writing–review and editing (equal).

## FUNDING INFORMATION

This study was performed as part of a PhD program for the lead author (JP). Funding for the PhD was provided by University Hospital Limerick and the Corresponding Author at the University of Limerick.

## CONFLICT OF INTEREST

The authors certify that they have no affiliations with or involvement in any organisation or entity with any financial interest, or non‐financial interest in the subject matter or materials discussed in this manuscript that would constitute a conflict of interest.

## AUTHORS' CONSENT

All the authors have revised the manuscript critically and have approved the final draft.

## Supporting information


Appendix S1

## Data Availability

The data that support the findings of this study are available from the corresponding author upon reasonable request.
