# Supplementary material for: Dermatology mycology diagnostics in Ireland: National deficits identified in 2022 that are relevant internationally
Source: Mycoses. 2022 Dec 9;66(3):249–57. doi: 10.1111/myc.13549 (PMC10107536; doi:10.1111/myc.13549)
Supplement: Supplementary file 1 — Appendix S1 [file MYC-66-249-s001.docx]

|  | ULHG | Dermagenius® 2.0 | Dermagenius® 3.0 | EUROArray Dermatomycosis | Dermatophytes and Other Fungi | Novaplex^tm^ Dermatophyte |
| --- | --- | --- | --- | --- | --- | --- |
|  | No. Orgs | CE-IVD | CE-IVD | CE-IVD | CE-IVD | RESEARCH USE ONLY |
|  | 02 - 21 | Pathonostics® | Pathonostics® | EUROImmun | Ausdiagnostics | Seegene |
| Organism | No | Netherlands | Netherlands | Germany | Australia | South Korea |
| Arthroderma amazonicum | 0 | No Target | Dermatophyte ^b^ | Dermatophyte | No Target | No Target |
| Arthroderma ciferrii | 0 | No Target | Dermatophyte ^b^ | Dermatophyte | No Target | No Target |
| Arthroderma cuniculi | 0 | No Target | Dermatophyte ^b^ | Dermatophyte | No Target | No Target |
| Arthroderma curreyi | 0 | No Target | Dermatophyte ^b^ | Dermatophyte | No Target | No Target |
| Arthroderma eboreum | 0 | No Target | Dermatophyte ^b^ | Dermatophyte | No Target | No Target |
| Arthroderma flavescens | 0 | No Target | Dermatophyte ^b^ | Dermatophyte | No Target | No Target |
| Arthroderma gertleri | 0 | No Target | Dermatophyte ^b^ | Dermatophyte | No Target | No Target |
| Arthroderma gloriae | 0 | No Target | Dermatophyte ^b^ | Dermatophyte | No Target | No Target |
| Arthroderma insingulare | 0 | No Target | Dermatophyte ^b^ | Dermatophyte | No Target | No Target |
| Arthroderma lenticulare | 0 | No Target | Dermatophyte ^b^ | Dermatophyte | No Target | No Target |
| Arthroderma melis | 0 | No Target | Dermatophyte ^b^ | Dermatophyte | No Target | No Target |
| Arthroderma multifidum | 0 | No Target | Dermatophyte ^b^ | Dermatophyte | No Target | No Target |
| Arthroderma phaseoliforme | 0 | No Target | Dermatophyte ^b^ | Dermatophyte | No Target | No Target |
| Arthroderma quadrifidum | 0 | No Target | Dermatophyte | Dermatophyte | No Target | No Target |
| Arthroderma thuringiensis | 0 | No Target | Dermatophyte ^b^ | Dermatophyte | No Target | No Target |
| Arthroderma tuberculatum | 0 | No Target | Dermatophyte ^b^ | Dermatophyte | No Target | No Target |
| Arthroderma uncinatum | 0 | No Target | Dermatophyte | Dermatophyte | No Target | No Target |
| Arthroderma vespertilii | 0 | No Target | Dermatophyte ^b^ | Dermatophyte | No Target | No Target |
| Nannizzia corniculata | 0 | No Target | Dermatophyte ^b^ | Dermatophyte | No Target | No Target |
| Nannizzia duboisii | 0 | No Target | Dermatophyte ^b^ | Dermatophyte | No Target | No Target |
| Nannizzia fulva (M. fulvum) | 0 | No Target | Dermatophyte | Nannizzia fulva | No Target | No Target |
| Nannizzia gypsea (M. gypseum) | 4 | No Target | Nannizia gypsea | Nannizzia gypsea | Nannizzia gypsea | No Target |
| Nannizzia incurvata (M. Incurvatum) | 0 | No Target | Dermatophyte | Nannizzia incurvata | No Target | No Target |
| Nannizzia nana | 0 | No Target | Dermatophyte ^b^ | Dermatophyte | No Target | No Target |
| Nannizzia persicolor (M. persicolor) | 3 | No Target | Dermatophyte | Nannizzia persicolor | No Target | No Target |
| Nannizzia praecox | 0 | No Target | Dermatophyte ^b^ | Dermatophyte | No Target | No Target |

Supplementary Table 1. Dermatophytes (part 1: Arthroderma and Nannizzia).

“No. Orgs”: The number of detections from skin, nail and hair samples in UL Hospitals Group from 2002 – 2021.

“XR”: Cross Reaction (Detection of organism via a target for another organism or complex of organisms)

^a^Organisms omitted from the inclusion tallies for Table 1

^b^Organism detection declared by manufacturer but not specifically listed in the analytical sensitivity report in the “Instructions For Use” document.

|  | ULHG | Dermagenius® 2.0 | Dermagenius® 3.0 | EUROArray Dermatomycosis | Dermatophytes and Other Fungi | Novaplex^tm^ Dermatophyte |
| --- | --- | --- | --- | --- | --- | --- |
|  | No. Orgs | CE-IVD | CE-IVD | CE-IVD | CE-IVD | RESEARCH USE ONLY |
|  | 02 - 21 | Pathonostics® | Pathonostics® | EUROImmun | Ausdiagnostics | Seegene |
| Organism | No | Netherlands | Netherlands | Germany | Australia | South Korea |
| Trichophyton balcaneum | 0 | No Target | Dermatophyte ^b^ | Dermatophyte ^b^ | Trichophyton spp. ^b^ | Trichophyton rubrum Complex |
| Trichophyton benhamiae | 0 | Trichophyton benhamiae | Trichophyton benhamiae | Trichophyton benhamiae | T. mentagrophytes complex ^b^ | T. mentagrophytes Complex |
| Trichophyton bullosum | 0 | No Target | Dermatophyte ^b^ | Trichophyton bullosum | Trichophyton spp. ^b^ | T. mentagrophytes complex XR |
| Trichophyton circonvolutum | 0 | No Target | Dermatophyte ^b^ | Dermatophyte ^b^ | Trichophyton spp. ^b^ | Trichophyton rubrum Complex |
| Trichophyton concentricum | 0 | T. benhamiae XR | T. benhamiae XR | T. concentricum/erinacei | Trichophyton spp. ^b^ | T. mentagrophytes complex XR |
| Trichophyton equinum | 0 | T. tonsurans XR | Dermatophyte ^b^ | Trichophyton equinum | Trichophyton spp. ^b^ | T. mentagrophytes complex XR |
| Trichophyton erinacei | 0 | T. benhamiae XR | T. benhamiae XR | Trichophyton erinacei | T. mentagrophytes complex ^b^ | T. mentagrophytes Complex |
| Trichophyton eriotrephon | 0 | No Target | Dermatophyte ^b^ | Trichophyton eriotrephon | Trichophyton spp. ^b^ | T. mentagrophytes complex XR |
| Trichophyton gourvilii | 0 | No Target | Dermatophyte ^b^ | Dermatophyte ^b^ | Trichophyton spp. ^b^ | Trichophyton rubrum Complex |
| Trichophyton interdigitale | 200 | Trichophyton interdigitale | (T. interdigitale/mentagrophytes) | Trichophyton interdigitale | T. mentagrophytes Complex ^b^ | T. mentagrophytes Complex |
| Trichophyton kuryangei | 0 | No Target | Dermatophyte ^b^ | Dermatophyte ^b^ | Trichophyton spp. ^b^ | Trichophyton rubrum Complex |
| Trichophyton mentagrophytes | 524 | Trichophyton mentagrophytes | (T. interdigitale/mentagrophytes) | Trichophyton mentagrophytes | T. mentagrophytes Complex ^b^ | T. mentagrophytes Complex |
| T. mentagrophytes complex | 0 | N/A | N/A | N/A | T. mentagrophytes Complex | N/A |
| Trichophyton mentagrophytes | 0 | T. mentagrophytes XR | T. schoenleinii/quinckeanum | Trichophyton quinckeanum | T. mentagrophytes Complex ^b^ | T. mentagrophytes Complex |
| Trichophyton rubrum | 1205 | (T. rubrum /soudanense) | (T. rubrum /soudanense) | Trichophyton rubrum | T. rubrum complex ^b^ | Trichophyton rubrum Complex |
| Trichophyton rubrum complex | 0 | N/A | N/A | N/A | T. rubrum complex | N/A |
| Trichophyton schoenleinii | 3 | T. mentagrophytes XR | T. schoenleinii/quinckeanum | Trichophyton schoenleinii | Trichophyton spp. ^b^ | T. mentagrophytes complex XR |
| Trichophyton simii | 0 | No Target | Dermatophyte ^b^ | Trichophyton simii | Trichophyton spp. ^b^ | T. mentagrophytes complex XR |
| Trichophyton soudanense | 2 | (T. rubrum /soudanense) | (T. rubrum /soudanense) | Trichophyton soudanense | T. rubrum complex ^b^ | Trichophyton rubrum Complex |
| Trichophyton species^a^ | 14 | N/A | Dermatophyte ^b^ | N/A | Trichophyton spp. | N/A |
| Trichophyton tonsurans | 111 | Trichophyton tonsurans | Trichophyton tonsurans | Trichophyton tonsurans | Trichophyton spp. ^b^ | T. tonsurans/rubrum complex |
| Trichophyton verrucosum | 35 | Trichophyton verrucosum | Trichophyton verrucosum | T. verrucosum/eriotrephon | Trichophyton spp. ^b^ | T. mentagrophytes complex XR |
| Trichophyton violaceum | 12 | Trichophyton violaceum | Trichophyton violaceum | Trichophyton violaceum | T. rubrum complex ^b^ | Trichophyton rubrum Complex |
| Trichophyton yaoundei | 0 | No Target | Dermatophyte ^b^ | Dermatophyte ^b^ | Trichophyton spp. ^b^ | Trichophyton rubrum Complex |

Supplementary Table 2. Dermatophytes (part 2: Trichophyton species).

“No. Orgs”: The number of detections from skin, nail and hair samples in UL Hospitals Group from 2002 – 2021.

“XR”: Cross Reaction (Detection of organism via a target for another organism or complex of organisms)

^a^Organisms omitted from the inclusion tallies for Table 1

^b^Organism detection declared by manufacturer but not specifically listed in the analytical sensitivity report in the “Instructions For Use” document.

|  | ULHG | Dermagenius® 2.0 | Dermagenius® 3.0 | EUROArray Dermatomycosis | Dermatophytes and Other Fungi | Novaplex^tm^ Dermatophyte |
| --- | --- | --- | --- | --- | --- | --- |
|  | No. Orgs | CE-IVD | CE-IVD | CE-IVD | CE-IVD | RESEARCH USE ONLY |
|  | 02 - 21 | Pathonostics® | Pathonostics® | EUROImmun | Ausdiagnostics | Seegene |
| Ctenomyces serratus | 0 | No Target | Dermatophyte ^b^ | Dermatophyte | No Target | No Target |
| Epidermophyton floccosum | 28 | Epidermophyton floccosum | Epidermophyton floccosum | Epidermophyton floccosum | Epidermophyton floccosum | Epidermophyton floccosum |
| Lophophyton gallinae | 0 | No Target | Dermatophyte ^b^ | Dermatophyte | No Target | No Target |
| Microsporum audouinii | 4 | Microsporum audouinii | Microsporum audouinii | Microsporum audouinii | Microsporum species ^b^ | Microsporum species |
| Microsporum canis | 113 | Microsporum canis | Microsporum canis | Microsporum canis | Microsporum canis | Microsporum species |
| Microsporum ferrugineum | 2 | M. canis XR | M. canis XR | Microsporum ferrugineum | Microsporum species ^b^ | Microsporum species |
| Microsporum species | 0 | N/A | N/A | N/A | Microsporum species | N/A |
| Paraphyton cookei | 0 | No Target | Dermatophyte ^b^ | Dermatophyte | No Target | No Target |
| Paraphyton mirabile | 0 | No Target | Dermatophyte ^b^ | Dermatophyte | No Target | No Target |

Supplementary Table 3. Dermatophytes (part 3: other species).

“No. Orgs”: The number of detections from skin, nail and hair samples in UL Hospitals Group from 2002 – 2021.

“XR”: Cross Reaction (Detection of organism via a target for another organism or complex of organisms)

^a^Organisms omitted from the inclusion tallies for Table 1

^b^Organism detection declared by manufacturer but not specifically listed in the analytical sensitivity report in the “Instructions For Use” document.

|  | ULHG | Dermagenius® 2.0 | Dermagenius® 3.0 | EUROArray Dermatomycosis | Dermatophytes and Other Fungi | Novaplex^tm^ Dermatophyte |
| --- | --- | --- | --- | --- | --- | --- |
|  | No. Orgs | CE-IVD | CE-IVD | CE-IVD | CE-IVD | RESEARCH USE ONLY |
|  | 02 - 21 | Pathonostics® | Pathonostics® | EUROImmun | Ausdiagnostics | Seegene |
| Organism | No | Netherlands | Netherlands | Germany | Australia | South Korea |
| Absidia species | 1 | No Target | No Target | No Target | No Target | No Target |
| Acremonium species | 11 | No Target | No Target | No Target | No Target | No Target |
| Alternaria alternata | 2 | No Target | No Target | No Target | No Target | No Target |
| Alternaria species | 1 | No Target | No Target | No Target | No Target | No Target |
| Aspergillus candidus | 1 | No Target | No Target | No Target | No Target | No Target |
| Aspergillus clavatus | 0 | No Target | No Target | No Target | Aspergillus spp. ^b^ | No Target |
| Aspergillus flavus | 0 | No Target | No Target | No Target | Aspergillus spp. ^b^ | No Target |
| Aspergillus fumigatus | 27 | No Target | No Target | No Target | Aspergillus spp. ^b^ | No Target |
| Aspergillus giganteus | 0 | No Target | No Target | No Target | Aspergillus spp. ^b^ | No Target |
| Aspergillus glaucus | 8 | No Target | No Target | No Target | No Target | No Target |
| Aspergillus nidulans | 10 | No Target | No Target | No Target | No Target | No Target |
| Aspergillus niger | 6 | No Target | No Target | No Target | Aspergillus spp. ^b^ | No Target |
| Aspergillus ochraceus | 1 | No Target | No Target | No Target | No Target | No Target |
| Aspergillus spp. | 0 | No Target | No Target | No Target | Aspergillus spp. | No Target |
| Aspergillus terreus | 33 | No Target | No Target | No Target | No Target | No Target |
| Aspergillus versicolor | 9 | No Target | No Target | No Target | No Target | No Target |
| Chrysosprorum keratinophilum | 2 | No Target | No Target | No Target | No Target | No Target |
| Cladophialophora Species | 1 | No Target | No Target | No Target | No Target | No Target |
| Fusarium solani | 0 | No Target | No Target | Fusarium solani | No Target | No Target |
| Fusarium oxysporum | 1 | No Target | No Target | Fusarium oxysporum | No Target | No Target |
| Fusarium species | 26 | No Target | No Target | No Target | No Target | No Target |
| Geotrichium species | 1 | No Target | No Target | No Target | No Target | No Target |
| Malassezia furfur | 17 | No Target | No Target | No Target | No Target | No Target |
| Moisissures species | 2 | No Target | No Target | No Target | No Target | No Target |
| Neoscytalidium dimidiatum | 4 | No Target | No Target | No Target | No Target | No Target |
| Onychocola canadensis | 1 | No Target | No Target | No Target | No Target | No Target |
| Paecilomyces sp | 1 | No Target | No Target | No Target | No Target | No Target |
| Penicillium species | 2 | No Target | No Target | No Target | No Target | No Target |
| Rhodotorula rubra | 1 | No Target | No Target | No Target | No Target | No Target |
| Rhodotorula species | 28 | No Target | No Target | No Target | No Target | No Target |
| Scedosporium apiospermum | 2 | No Target | No Target | No Target | No Target | No Target |
| Scopulariopsis brevicaulis | 122 | No Target | Scopulariopsis brevicaulis | Scopulariopsis brevicaulis | No Target | No Target |
| Scopulariopsis species | 1 | No Target | No Target | No Target | Scopulariopsis species | No Target |
| Trichosporon species | 28 | No Target | No Target | No Target | No Target | No Target |

Supplementary Table 4. Non-Dermatophyte Moulds.

“No. Orgs”: The number of detections from skin, nail and hair samples in UL Hospitals Group from 2002 – 2021.

^b^Organism detection declared by manufacturer but not specifically listed in the analytical sensitivity report in the “Instructions For Use” document.

|  |  | ULHG | Dermagenius® 2.0 | Dermagenius® 3.0 | EUROArray Dermatomycosis | Dermatophytes and Other Fungi | Novaplex^tm^ Dermatophyte |  |
| --- | --- | --- | --- | --- | --- | --- | --- | --- |
|  |  | No. Orgs | CE-IVD | CE-IVD | CE-IVD | CE-IVD | RESEARCH USE ONLY |  |
|  |  | 02 - 21 | Pathonostics® | Pathonostics® | EUROImmun | Ausdiagnostics | Seegene |  |
| Org Type | Organism | No | Netherlands | Netherlands | Germany | Australia | South Korea |  |
| Candida | Candida albicans | 129 | Candida albicans | Candida albicans | Candida albicans | Candida albicans | Candida albicans |  |
| Candida | Candida famata | 67 | No Target | No Target | No Target | No Target | No Target |  |
| Candida | Candida glabrata | 4 | No Target | No Target | No Target | Candida glabrata​ | No Target ^c^ |  |
| Candida | Candida guilliermondii | 82 | No Target | No Target | Candida guilliermondii | C. guilliermondii | No Target |  |
| Candida | Candida kefyr | 2 | No Target | No Target | No Target | No Target | No Target |  |
| Candida | Candida krusei | 11 | No Target | No Target | No Target | No Target | No Target ^c^ |  |
| Candida | Candida lusitaniae | 3 | No Target | No Target | No Target | No Target | No Target ^c^ |  |
| Candida | Candida parapsilosis | 139 | No Target | Candida parapsilosis | Candida parapsilosis | Candida parapsilosis | No Target ^c^ |  |
| Candida | Candida pelliculosa | 1 | No Target | No Target | No Target | No Target | No Target |  |
| Candida | Candida sake | 1 | No Target | No Target | No Target | No Target | No Target |  |
| Candida | Candida species^a^ | 87 | N/A | N/A | N/A | No Target | N/A |  |
| Candida | Candida spp Not albicans^a^ | 25 | No Target | N/A | N/A | No Target | N/A |  |
| Candida | Candida tropicalis | 16 | No Target | No Target | No Target | No Target | No Target ^c^ |  |
| Cryptococcus | Cryptococcus albidus | 1 | No Target | No Target | No Target | No Target | No Target |  |
| Cryptococcus | Cryptococcus laurentii | 1 | No Target | No Target | No Target | No Target | No Target |  |
| Cryptococcus | Cryptococcus neoformans | 2 | No Target | No Target | No Target | No Target | No Target |  |
| Resistance | SQLE mutations ^d^ | 0 | No Target | SQLE mutations ^d^ | No Target | No Target | No Target |  |

Supplementary Table 5. Yeast and other targets. SQLE mutations are associated with terbinafine resistance.

“No. Orgs”: The number of detections from skin, nail and hair samples in UL Hospitals Group from 2002 – 2021.

^c^targets are available on another PCR kit from this manufacturer (not validated for dermatological specimens).

^d^ SQLE mutations are associated with terbinafine resistance.
